# Insulin-Like Growth Factor-1 Signaling in Lung Development and Inflammatory Lung Diseases

**DOI:** 10.1155/2018/6057589

**Published:** 2018-06-19

**Authors:** Zheng Wang, Wenting Li, Qiongya Guo, Yuming Wang, Lijun Ma, Xiaoju Zhang

**Affiliations:** ^1^Department of Respiratory and Critical Medicine, Zhengzhou University People's Hospital, Zhengzhou 450003, China; ^2^Department of Infectious Diseases, Anhui Provincial Hospital, Hefei 230001, China; ^3^Department of Digestive Diseases, Zhengzhou University People's Hospital, Zhengzhou 450003, China; ^4^Medical Research Center, Zhengzhou University People's Hospital, Zhengzhou 450003, China

## Abstract

Insulin-like growth factor-1 (IGF-1) was firstly identified as a hormone that mediates the biological effects of growth hormone. Accumulating data have indicated the role of IGF-1 signaling pathway in lung development and diseases such as congenital disorders, cancers, inflammation, and fibrosis. IGF-1 signaling modulates the development and differentiation of many types of lung cells, including airway basal cells, club cells, alveolar epithelial cells, and fibroblasts. IGF-1 signaling deficiency results in alveolar hyperplasia in humans and disrupted lung architecture in animal models. The components of IGF-1 signaling pathways are potentiated as biomarkers as they are dysregulated locally or systemically in lung diseases, whereas data may be inconsistent or even paradoxical among different studies. The usage of IGF-1-based therapeutic agents urges for more researches in developmental disorders and inflammatory lung diseases, as the majority of current data are collected from limited number of animal experiments and are generally less exuberant than those in lung cancer. Elucidation of these questions by further bench-to-bedside researches may provide us with rational clinical diagnostic approaches and agents concerning IGF-1 signaling in lung diseases.

## 1. Introduction

Insulin-like growth factors (IGFs) are produced mainly by the liver cells in response to pituitary hormones and form a feedback signaling loop with pituitary, liver, and growth hormone-releasing hormone release by the hypothalamus [1, 2]. The activity of IGFs is approximately 1% that of insulin with respect to the metabolism of glucose, lipid, and protein. IGFs also promote differentiation of myoblastic or osteoblastic tissues into muscle and bone [3]. In addition to its role as a mediator in metabolism, IGF-1 is also implicated in developmental disorders, a variety of diseases other than metabolic disorders, or cancers. Immunological and genetic analyses have affirmed the expression of IGF-1 signaling components in cells from the normal lung tissue, including airway cells, lung parenchymal cells, smooth muscle cells, lung fibroblasts, and alveolar macrophages [4]. Recent researches redefine the previously recognized role of IGF-1 signaling in lung development and diseases, such as congenital disorders, cancers, inflammation, and fibrosis [5]. Abnormal IGF-1/IGF-1R signaling has been extensively studied in lung cancer that mediates oncogenesis, progression, metastasis, resistance to chemotherapy, or tyrosine kinase inhibitors (TKIs) [6]. The role of IGF-1/IGF-1R signaling abnormalities in lung cancer has been extensively reviewed elsewhere [2, 7–11]. Clinical trials have been finished or just begun to investigate the safety and efficacy of antibodies against IGF-1 signaling in lung cancer [12–16]. The roles of IGF-1 signaling are also documented in patients with pneumothorax and alveolar rhabdomyosarcoma (ARMS), in animal models of pulmonary artery hypertension and postpneumonectomy lung regeneration [5, 17–19]. In this review, we focus mainly on the roles of IGF-1 signaling in lung development and inflammatory diseases, as will be discussed in detail below.

## 2. IGF Signaling Pathway

IGF signaling pathway is composed of three ligands (IGF-1, IGF-2, and insulin), three receptors [IGF-1 receptor (IGF-1R), IGF-2R, and insulin receptor (IR)], and a superfamily of six IGF binding proteins (IGFBPs 1–6) [20]. Insulin-like growth factor-1 (IGF-1) and IGF-2 are the only two members of IGFs identified hitherto. IGFs were firstly discovered by Salmon Jr. and Daughaday as single chain polypeptide hormones in their pursuit for mediators of the activity of growth hormone in 1957 [21]. IGF-1 and IGF-2 share 50% homology, as well as some functions with insulin in regulating metabolism and growth. IGF-2 (also known as IGF-II or somatomedin A) is a 67-amino-acid peptide, and combination of IGF-2 with IGF-2R has not been found to associate with intracellular signaling. IGF-1 (also known as IGF-I or somatomedin C) is a 7.5 kDa peptide that has 70-amino-acid residues and four domains. IGF-1 gene is located on the long arm of chromosome 12 (12q23.2) and consists of 6 exons. The prototype of IGF-1 protein (pro-IGF-1) contains a C-terminal peptide that is proteolytically processed in the Golgi apparatus before secretion. IGF-1 gene splicing generates three splice isoforms, IGF-1Ea, IGF-1Eb, and IGF-1Ec. The functional difference among these isoforms has not been extensively analyzed in other cells and organs [22].

**Figure 1:**
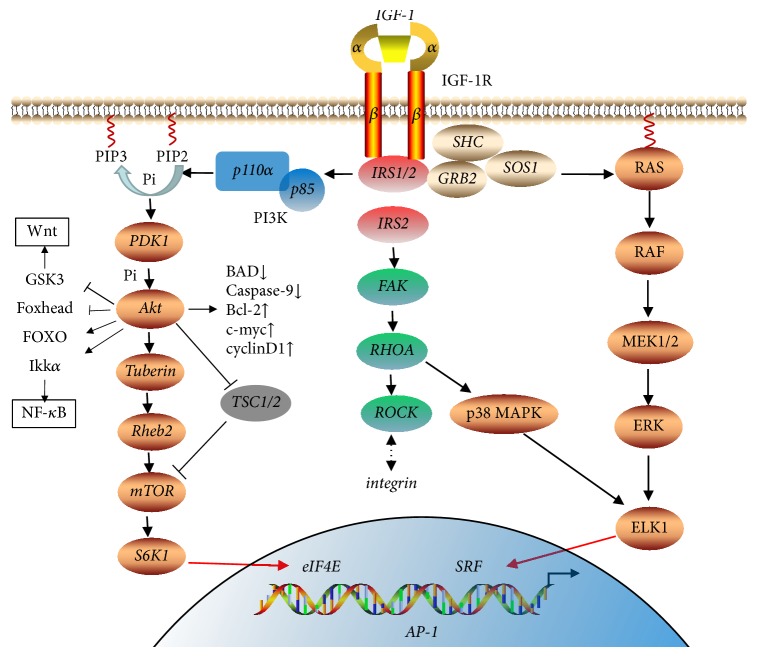
IGF signaling pathway. IGF-1 binds with IGF-1R and activates its intracellular kinase activity, which activates insulin receptor substrates (IRS-1 and IRS-2). IRS-1 and IRS-2 further regulate the transcription of downstream genes as well as cell physiology by activating RAS/Raf/MAPK and PI3K/Akt/mTOR signaling pathways. IRS-2 also activates Rho-kinase (ROCK) and p38 MAPK via focal adhesion kinase (FAK) and crosstalk with Wnt, NF-*κ*B, and integrin signaling pathways.

IGF-1, IGF-2, or insulin binds with different receptors or the receptor hybrids [23]. Alternative splicing of exon 11 gives rise to two splice variant isoforms of IR, IRA, and IRB, which may form IGF-1R/IR heterodimer with IGF-1R. Although IGF-1 may also combines with IRA or IGF-1R/IRA dimer, it combines with IGF-1R in most situations. IGF-1R is a type 2 tyrosine kinase transmembrane receptor that is normally found as a heterotetramer with two extracellular *α* and two membrane-spanning *β* subunits [23, 24]. Ligands bind to the *α* subunit and activate the intrinsic tyrosine kinase activity of the *β* subunit, which further binds and activates insulin receptor substrates (IRS) and Shc. Phosphorylation of IRS1 or IRS2 further activates the PI3K-Akt-mTOR signaling pathway via binding to p85 regulatory subunit of PI3K. Shc interacts with the growth factor receptor bound protein 2 (GRB2) and son-of-sevenless (SOS) to activate RAS, RAF, and the ERK/MAPK signaling pathway. IRS2 may regulate cellular motility by modulation of integrin expression possibly via RHOA, focal adhesion kinase (FAK), and Rho-kinase (ROCK) [23, 24]. GSK-3, IKK*α*, Foxhead, FOXO, TSC1/2, BAD, caspase-9, Bcl-2, cyclinD1 are downstreaming IGF-1/PI3K/Akt signaling, which are essential for metabolism, cell cycle, apoptosis, Wnt, and NF-*κ*B signaling activation. MAPK signaling pathway is also the key controller of cell fate that includes cell adhesion, proliferation, apoptosis, and survival [20, 23, 24]. The IGF-1 signaling pathway is depicted in Figure 1.

IGFBPs bind with high affinity with IGF-1 or IGF-2 and less affinity with insulin. Approximately 98% of IGF-1 in the body fluid is bound to one of six IGFBPs, which may prolong the half-life of circulating IGF-1. IGFBPs may inhibit or promote IGF-1/IGF-1R signaling activities, depending on the type of IGFBPs, organs, and cells [25]. IGFBPs may also exert biological functions independent of IGF ligands and receptors [26]. Regulation of IGF-1/IGF-1R signaling may happen at the posttranscriptional level by mechanisms involving RNA stabilization and protein acetylation [27–29]. Noncoding RNAs also mediate the bioactivity of IGF-1 and IGF-1R [30]. Expressions of IGF-1, IGF-1R, and PI3K, as well as phosphorylation of AKT and FoxO3a, are inhibited by miR-470, miR-669b, or miR-681 [30]. There lies extensive crosstalk between IGF-1 and other signaling pathways that control cell physiology, such as insulin, integrin, Wnt, and epidermal growth factor receptor (EGFR) signaling pathways [1, 7, 8, 11, 31, 32].

## 3. IGF-1 Signaling in Lung Development

### 3.1. Congenital IGF-1/IGF-1R Signaling Abnormalities and Lung Development

Individuals with congenital IGF-1 signaling deficiencies are born predominantly with metabolic and systemic developmental alterations, less with lung disorders [33, 34]. Lung hypoplasia may manifest in patients losing one IGF-1R gene copy for chromosome 15 deletion, along with other disorders such as intrauterine growth retardation (IUGR), microcephaly, abnormal face and ears, micrognathia, highly arched palate, renal abnormalities, failure to thrive, developmental delay, and mental retardation [35]. Mutations of IGF-1 gene result in decreased serum levels or binding affinity of IGF-1; patients are characterized by severe intrauterine growth retardation, microcephaly, postnatal growth failure, severe psychomotor retardation, sensorineural deafness, and mild dysmorphic features [36–38]. Primary growth hormone insensitivity (Laron syndrome) is the most common and prototypical condition within the primary IGF deficiency (PIGFD) category, which is characterized by extremely low serum IGF-1 levels that may require IGF-1 treatment [39]. Patients with Laron syndrome have abnormal phenotype of the growth factor receptor (mutated or inactivated), who demonstrate growth and organic development retardation, short stature, delayed bone age, and reduced exercise capacity, but their lung function is normal, and none of lung injury or bronchopulmonary dysplasia has been reported [40]. Acromegaly may present with high levels of growth hormone and varying levels of IGF-1 and similar lung structure and functions compared with health controls, and none of the lung functional, radiological, and biological findings correlated with IGF-1 levels [41, 42].

Interestingly, the velocity of intrauterine and postnatal growth is correlated with levels of IGF-1. Prenatal tracheal occlusion or ligation, a well-documented stimulator of fetal lung growth, is accompanied with an increase in expression of IGF-1 within rat lungs [43]. Neonatal cord plasma levels of IGF-1 and IGFBP-3 are associated with fetal growth and possibly fetal lung growth and are decreased in the babies of mothers who had smoked [44, 45]. Prenatally parental cigarette smoking in female mice reduces the mRNA levels of Igf1 and Igf1r and gives rise to CpG-site specific methylation changes in Igf1r [46]. Decreased level of IGF-1 is responsible for hypoxia-induced abnormal development and chronic inflammation of the lungs in postnatal rats [47]. This might be supported by large-scale epidemiological studies, which show the positive linear associations between plasma IGF-1 and lung function, most evidently in men and in women above 50 years old [48, 49].

### 3.2. IGF-1 Signaling Deficiency Models

IGF-1 and IGF-1R are widely distributed throughout the neonatal rodent lungs. The impact on the lung development is more prominent in animals than in humans of IGF-1 or IGF-1R deficiency [1]. Studies with animal models suggest that IGF-1 modulates the development and differentiation of many types of lung cells, including airway basal cells, club cells, alveolar epithelial cells, and fibroblasts. The mechanisms involved remain largely unknown, but it is likely that autocrine and paracrine effects are involved, possibly via downstream and interacting signaling pathways of IGF-1.

Plasma IGF-1 is elevated or decreased in growth hormone transgenic or growth hormone receptor knockout mice. Except for that mice are less viable, lungs of survivors have well-developed bronchi, bronchioles with Clara cells and alveoli, and no signs of inflammation [50, 51]. Igf1 transgene mice show alveolar hyperplasia [52]. Prenatal Igf1−/− mutant mice are less viable for lethal neonatal respiratory distress, which displayed immature and delayed distal pulmonary organogenesis as manifested by severe lung hypoplasia, thickened mesenchyme, diffuse extracellular matrix deposition, thinner smooth muscles, and dilated blood vessels [53]. Similarly, IGF-1R−/− (knockout) embryos display active cell proliferation and apoptosis, severe lung hypoplasia, and markedly underdeveloped diaphragms and could hardly survive lethal neonatal respiratory distress [53–55]. In contrast, IGF-1Rneo/− mice that express 22% of normal IGF-1R levels are viable and have normal lung histomorphometric characteristics and normal breathing response to hypercapnia [55]. The discrepancies between IGF-1 and IGF-1R deficiency may be attributed to the universality of IGF-1R in inducing signaling or different compensatory responses [56]. Subsequent alterations of RNA transcriptome in IGF-1 knockout include genes related to vascularization, morphogenesis, cellular growth, immunological reactions, and MAPK, Wnt, and cell adhesion pathways [57]. Notably, MAPK/ERK2 is activated while Egr1 and Ctgf are inactivated [57]. These genes and signaling pathways may serve as mediators downstreaming IGF-1 signaling in epithelium maturation during prenatal lung development [57].

Human airway basal cells (BC) function as stem/progenitor cells of the human airway epithelium, capable of differentiating into ciliated and secretory cells during turnover and repair. Studies have revealed that IGF-1 signaling mediates the developmental fate of basal cell differentiation, possibly via FoxO-mediated p63 inhibition [58]. Knockout of histone methyltransferase Ezh2 derepresses IGF-1 signaling, which results in reduced lung volume, perturbed airway lineage specification, and a defect in alveoli formation in mice [54]. IGF-1 signaling* ex vivo* in wild-type lungs induces dilatation of airways, basal cell differentiation, and expression of Krt5, Krt14, and Trp63 mRNA [59]. These results suggest that repression of Igf1 expression by Ezh2 in lung epithelial cells is likely to be a crucial process for controlling basal cell fate determination and maintaining a proper control of lineage specification during embryonic lung morphogenesis. The role of IGF-1 signaling in basal cell differentiation is further indicated in a coculture system of human airway basal cells and lung microvasculature endothelial cells. Differentiation of basal cells is stimulated by coculture via activation of IGF-1R-mediated Akt and ERK1/2 signaling and is suppressed by siRNA-mediated knockdown of IGF-1R in basal cells [60].

IGF-1R deficiency disturbed airway epithelial differentiation in adult mice and enhanced proliferation and altered morphology in distal airway club cells [61]. These morphological alterations are accompanied by increased expression in epithelial cell precursor-specific genes and reduced expression of Clara cell secretory protein (CCSP) [61]. The expression of IGF-1R mRNA and JNK protein is elevated, and ERK signaling is repressed after club cell ablation with naphthalene challenge [61]. Blockage of IGF-1, or of IGF-1R with a truncated soluble IGF-1R, reduces lung tissue fraction, lung procollagen I and elastin fiber production, total lung cell and secondary crest cell DNA synthesis, and lung small vessel density and impairs alveologenesis, as assessed by secondary crest formation and mean linear intercepts [62]. The expression of IGF-1 mRNA is upregulated during the transformation of cultured alveolar type II (ATII) cells to alveolar type I (ATI) cells [63], and addition of antibodies against IGF-1 or IGF-1R inhibits ATII cell proliferation and trans-differentiation to some extent [64]. Recombinant IGF-1 promotes the differentiation of ATII to ATI, which is abrogated by a IGF-1R blocker, and is accompanied by activation of Wnt5a, protein kinase C, and *β*-catenin, suggesting the involvement of Wnt5a-dependent activation of Wnt/Frizzled signaling pathway [63].

IGF-1 collaborates with leukemia inhibitory factor (LIF) in lung alveolar epithelium and vascular maturation [65]. IGF-1-deficient mouse embryos display a higher proportion of type II pneumocytes, less differentiated type I pneumocytes, and failure in alveolar capillary remodeling compared to wild-type mice [65]. Lif/Igf1 double knockout lungs show aggravated pulmonary hypoplasia, lower airway volume, increased proliferation, collapsed alveoli that are lined by ATII cells, and elevated levels of ERK1/2 activation [65]. Fibroblast growth factor-stimulated IGF-1 signaling may also contribute to alveolar elastogenesis in a paracrine manner [66].

### 3.3. IGF-1 Signaling in Bronchopulmonary Dysplasia (BPD)

Bronchopulmonary dysplasia (BPD), characterized by decreased alveolar septation and/or lung inflammations, is the most common chronic lung sequelae that occurs in survivors of very preterm birth or of intensive care after birth [67]. Hyperoxia-disturbed IGF-1 signaling is believed to play an important role in BPD [67, 68]. Serum levels of IGF-1 are significantly decreased and low serum IGF-1 levels at 33 weeks' postmenstrual age predict high risk for developing BPD [69, 70]. However, the expression of IGF-1 in epithelial lining fluid, epithelial cells, and peribronchial myofibroblasts was increased in BPD [71, 72]. This is further identified in hyperoxia-stimulated* ex vivo* neonatal rat lung model, as IGF-1 staining is most abundant in airway and alveolar epithelial cells and cell proliferation is accompanied by excessive IGF-1 mRNA and protein expression and could be inhibited by IGF-1 antibody [73].

Hyperoxia inactivates IGF-1/IGF-1R signaling by attenuating their specific binding affinity in neonatal lungs or fetal lung distal epithelial cells, which further impairs lung cell proliferation, secondary crest formation, and alveologenesis [74]. The proteins and mRNA of IGF-1 and IGF-1R increase during hyperoxia lung injury and are highly abundant in ATII cells. Among IGFBPs, IGFBP-2, IGFBP-3, and IGFBP-5 mRNA increased during hyperoxia-induced injury, and IGFBP-5 mRNA is also elevated during the recovery phase [74]. Hyperoxia significantly increases IGF-1 levels in BALF and mouse lung lysates, as well as IGF-1R-positive neutrophils in peripheral blood and in BALF [75]. IGF-1 induced a dose-dependent chemotaxis of polymorphonuclear cells to the alveolar space, which is decreased by systemic pretreatment of an IGF-1R antibody in hyperoxia-injured mice [75]. The ratio of epithelial lining fluids concentrations of IGF-1 to PAPP-A (pregnancy-associated plasma protein-A), a metalloproteinase that dissociates IGFBPs, is increased and associated with lung injury and malformation in BPD [76]. Another research identifies the role and therapeutic potentials of miR-489-mediated IGF-1 expression in hyperoxia-exposed lungs or BPD [77]. MiR-489 is reduced in BPD infants and in a mice model of hyperoxia-induced BPD, in which hyperoxia increases IGF-1 expression in mice lungs. Inhibition of miR-489 permits alveolar septation to proceed. MiR-489 is of epithelial origin and presents in exosomes, while its targets Igf1 and Tnc are produced by fibroblasts, suggesting the presence of paracrine effecting mechanisms [77]. Hyperoxia exposure can promote pneumocyte apoptosis and inhibit the expression of CCSP [78]. Intraperitoneal injection of recombinant human insulin-like growth factor-1 (rhIGF-1) alleviates pneumocyte apoptosis, restores CCSP expression, and removes the block of the formation of lung alveoli in hyperoxia-exposed rats, which provides rationale for clinical application of rhIGF-1 in prevention and treatment of BPD [78]. Decrease in IGF-1R expression protects mice against 90% oxygen-induced lung injury, as demonstrated by less edema, vascular extravasation, and respiratory failure than control mice lungs [79]. In a clinical trial investigating the efficacy of rhIGF-I/rhIGFBP-3 in prevention of retinopathy of prematurity (ROP) in premature infants, the occurrence of BPD serves as a secondary outcome [80]. The rhIGF-I/rhIGFBP-3 seems to reduce the occurrence of BPD as compared with standard care: 21.3% (10 in 47 infants) versus 44.9% (22 in 49 infants) [80]. These findings in all demonstrate the therapeutic potentials of IGF-1 in BPD, which possibly restores hyperoxia-disrupted regeneration and differentiation of alveolar epithelium. 

## 4. IGF-1 in Fibrotic Lung Diseases

### 4.1. Evidences of IGF-1 Signaling in Fibrotic Lung Diseases

Upregulated IGF-1 expression and/or signaling is present in (1) patients with idiopathic fibrotic lung diseases, such as idiopathic pulmonary fibrosis (IPF) and late-stage sarcoidosis [81], (2) patients with secondary fibrosis, such as silicosis, pneumoconiosis, drug-induced pulmonary fibrosis, mustard gas-induced pulmonary fibrosis, systemic sclerosis-related interstitial lung disease, and rheumatoid arthritis-related interstitial lung disease [82–89], and (3) animal models of lung fibrosis induced by bleomycin, paraquat or silica challenge, and radiation [90–92]. The complexity of IGF-1 signaling is evidenced in fibrotic process, as it may be time- and space-dependent. The expression of IGF-1 mRNA is downregulated in lung tissues from patients with idiopathic pulmonary fibrosis (IPF) or sarcoidosis, while basal or TGF-*β*1-stimulated IGF-1 expression is higher in fibroblasts collected from IPF or fibrotic (Phase III or IV) sarcoidosis than health controls [93, 94]. An increase of IGF-1 and IGF-1R expression in alveolar macrophages (AM) and ATII cells is observed in IPF and sarcoidosis [95]. In lung tissues with late-stage IPF and those from normal controls, only alveolar macrophages contain IGF-1 protein [95]. IGF-1 protein is localized and expressed in alveolar macrophages, mononuclear phagocytes, fibroblasts, ATII cells, vascular endothelial cells, and vascular smooth muscle cells in lung tissues with early-stage IPF [96]. Another similar study shows the localization of IGF-1 in AMs, interstitial macrophages, ATI and ATII, and ciliated columnar epithelial cells, as compared to healthy controls in that IGF-1 is expressed mainly in AMs of IPF patients [96]. Microarray data also show increased IGF-1 gene expression in IPF patients [97]. The levels of IGF-1 are elevated in BALF collected from systemic sclerosis patients as compared with control subjects and increase more in systemic sclerosis patients with abnormal computed tomography, which stimulates fibroblast proliferation [85].

### 4.2. IGF-1 Signaling Activates Lung Fibroblasts and Myofibroblasts

Although the exact role and regulation of IGF-1 signaling in pulmonary fibrosis are awaiting for elucidation, previous studies have given some clues. IGF-1 activates lung fibroblasts, in preventing apoptosis, stimulating the proliferation, and enhancing elastin and collagen production [98, 99]. IGF-1 stimulates the proliferation and differentiation of lung fibroblasts into myofibroblasts, increases collagen synthesis, and protects myofibroblasts from apoptosis and thus may directly or indirectly increase the overall quantity of myofibroblast-produced extracellular matrix in the lungs [100, 101]. IGF-1 requires IGF-1R to prevent mouse embryo fibroblasts from TNF-*α*- or ECM detachment-induced apoptosis* in vitro *[102, 103]. Blockade of the IGF-1/IGF-1R pathway by an IGF-1R antibody induces fibroblast apoptosis and subsequent resolution of pulmonary fibrosis [104]. IGF-1 activates and increases migration of lung fibroblast, possibly via IRS2/PI3K/Akt signaling pathway [104]. The antiapoptotic effect of IGF-1 in fibroblasts might be mediated by ras, FOS, EGR-1, EGR-2, PI3K/Akt, and/or MAPK signaling activation and could be blocked by an antibody or siRNA targeting IGF-1R [99, 103, 105]. Further microarray analysis in IGF-1-stimulated mouse NIH-3T3 fibroblasts shows elevated expressions of EGR-1, Twist, Jun, integrin *α*5, IL-4R*α*, and IL-3R*α* genes, which may control cell proliferation, apoptosis, mitogenesis, and differentiation [106]. IGF-1 secretion from lung epithelium is promoted by downregulation of miR-130b-3p in IPF, which induces collagen I expression and enhanced the proliferation and migration ability of fibroblast [107].

### 4.3. IGF-1 Signaling Induces EMT of Alveolar Epithelial Cells

IGF-1 may induce epithelial-mesenchymal transition (EMT) of ATII cells, an important process forming fibroblast-like, ECM-producing cells in pulmonary fibrosis [108]. IGF-1 and IGFBP-5 expressions are significantly reduced in ATII cells in a rat model of bleomycin-induced pulmonary fibrosis [109]. This is accompanied by an increase in surface protein C (SPC) and *α*-SMA expression and their colocalization in ATII cells, which suggests the involvement of IGF-1 signaling in EMT of ATII cells during the process of lung injury and fibrosis [109]. IGF-1 induces MMP-2 and MMP-9 expression in alveolar epithelial cells through ERK signaling [110]. There is crosstalk between IGF-1 and integrin signaling pathways, which modulates cell physiology such as growth, adhesion, migration, and extracellular matrix (ECM) production [32, 111, 112]. In a mice model of bleomycin-induced pulmonary fibrosis, three miRNAs and IGFBP3 are decreased, while IGF-1, IGFBP-5, and 8 miRNAs are increased [113]. Further bioinformatic analysis reveals IGF-1 signaling as among the targets of the differentially expressed miRNAs [113]. Fra-2/AP-1 contribute to pulmonary fibrosis in IPF and animal models of pulmonary fibrosis, possibly through activation of profibrotic signals, including IGF-1 [114].

Expression of IGFBPs is elevated in lung (IGFBP-3, IGFBP-5) and in serum levels (IGFBP-1, IGFBP-2) in IPF patients [113, 115–117]. IGFBP-2 is associated with proliferation of lung alveolar epithelial cells [118] and is highly expressed in sputum of IPF patients [119]. Syndecan-2 and tenascin, profibrotic genes that are increased in lung tissues of patients with systemic sclerosis or IPF, are induced in fibroblasts by TGF-*β* in an IGFBP-3-dependent manner or by IGFBP-3 alone [120, 121]. IGFBP-5 gives rise to EMT in lung epithelial cells and stimulates lung fibroblasts to generate ECM [115, 122, 123]. These effects are mediated through IGF-1-dependent ERK1/2 activation or induced by binding of IGFBP-5 with individual ECM components including collagen, vitronectin, heparin, laminin, thrombospondin-1, plasminogen activator inhibitor-1, and osteopontin [124, 125].

### 4.4. IGF-1 and Alveolar Macrophages in Pulmonary Fibrosis

IGF-1 secreted by lung macrophages or ATII cells may act in a paracrine manner in alveolar compartment. IGF-1 released by alveolar macrophages (AMs) is elevated in BALF, which increases fibroblast mitogenic activity of sarcoidosis BALF that contributes to late-stage pulmonary fibrosis [126]. IGF-1 is released by quartz dust-exposed human macrophages as a result of phagocytosis* in vitro*, serving as a paracrine mitogen for human fibroblasts, ATII, and tracheobronchial epithelial cells, which is suggested to be involved in the epithelial repair and hyperplasia observed in silicosis [127, 128]. These stimulatory effects can be blocked by an IGF-1 antibody [128]. An IGF-1-dependent pathway is indicated for the stimulatory effect of rat ATII cells on type I collagen secretion by fibroblasts in the coculture system, which is blocked by an IGF-1 antibody [129].

As shown by Uh et al. in IPF patients, the number of IGF-1 positive interstitial macrophages correlates with the severity of collagen deposition and clinical impairment [96]. Mora et al. developed a mice model of progressive pulmonary fibrosis by infecting IFN-*γ*R-deficient mice with the murine gamma-herpesvirus 68 [130]. The coexpression of IGF-1 and fibronectin is elevated in AMs, which suggests the possible existence of alternative activated macrophage (M2 macrophage) [130, 131]. M2-polarization of macrophages could be induced by IL-4 and IGF-1 downstream signaling, including JNK and PI3K/AKT signaling pathways [132]. Alternatively activated macrophages secrete profibrotic growth factors including TGF-*β*, fibroblast growth factor (FGF), platelet-derived growth factor (PDGF*α*), which stimulates proliferation, ECM production, and differentiation of fibroblasts into myofibroblasts [133].

### 4.5. Feedback Loops of IGF-1 Signaling in Pulmonary Fibrosis

There may also exist positive feedback of IGF-1 signaling in the molecular levels that promote pulmonary fibrosis. Lung cells including activated AMs express both IGF-1 and IGF-1R, providing an autocrine activation effect [134, 135]. The expression of IGF-1 is promoted in IGF-1-stimulated lung fibroblast cells [99]. Overexpression of IGF-1 could be further magnified by exogenous TGF-*β*1 in fibroblasts recovered from BALF of IPF patients [92]. IL-4 receptor *α* and Twist, which are well-recognized profibrotic signals, are listed in upregulated genes in IGF-1-stimulated mouse fibroblasts [105]. On the other side, expression of IGF-1 is inhibited by Th1 cytokine IFN-*γ* and induced by Th2 cytokines IL-4 and IL-13, while pulmonary fibrosis is a typical Th2 response that IL-4 and IL-13 predominate [136, 137]. IL-4-induced, macrophage-derived IGF-1 protects myofibroblasts from apoptosis via Akt signaling, which may contribute to the persistence of myofibroblasts in the Th2-deviated environment of pulmonary fibrosis [138]. Interestingly, the relationship between IL-4 and IGF-1 is complicated, as IL-4 pretreatment restrains IGF-1-stimulated mitogenesis by ~60% in bovine fibroblasts via induction of IRS-2 phosphorylation and IL-4 receptor uses IRS-2 as its major substrate for tyrosine phosphorylation [139].

### 4.6. Evidences against IGF-1 as the Key Signaling Pathway in Pulmonary Fibrosis

Although abnormal IGF-1 signaling is implicated in human diseases and in animal models of lung fibrosis, there are also evidences suggesting that IGF-1 may not play a central role in the fibrotic process [140]. Adenoviral vector-mediated overexpression of IGF-1 in the mice lungs does not result in significant fibroblast or collagen accumulation featuring pulmonary fibrosis, but significant and prolonged inflammatory cell infiltration instead [141]. ATII cell-specific IGF-1 transgenic mice have normal lung pathology, cellularity of BALF, and total lung collagen content, as well as the extent of pulmonary inflammation or fibrosis compared with nontransgenic littermate controls [142]. Additionally, IGF-1 plus TGF-*β*1 transgene expression synergistically increased lung inflammation and collagen deposition compared with active TGF-*β*1 overexpression alone [143]. These data reveal that IGF-1 may not be able to initiate fibroproliferation on its own, but rather enhance pulmonary fibroproliferation in cooperation with TGF-*β*1. Another study shows that TGF-*β*1 but not IGF-1 increased *α*-SMA promoter activity, and no synergistic effect was seen with IGF-1 and TGF-*β*1 costimulation in mouse lung fibroblasts [144]. In addition, the role of IGF-1 in fibroblast activation may depend on the biomechanical properties of the ECM, as IGF-1 promotes *α*-SMA expression only in soft substrates while it enhances expression of other myofibroblast markers such as Col1a1 and Col3a1 in soft and stiff substrates [144]. Furthermore, chronic IGF-1 stimulation leads to mitochondrial dysfunction, decreased autophagy, and reduced cell viability in human fibroblasts* in vitro* [145]. The results altogether suggest that the profibrotic effect of IGF-1 depends on the time course of injury and repair as well as the properties of microenvironment and should be induced by convergence with TGF-*β*1 or other profibrotic signals.

**Figure 2:**
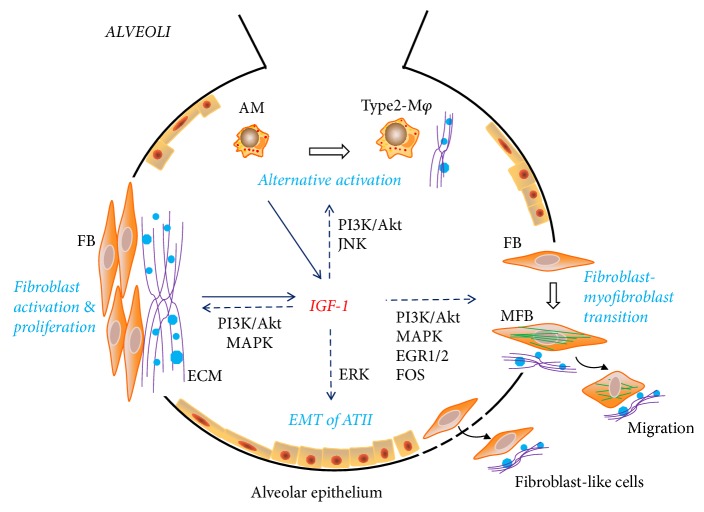
Cellular responses induced by IGF signaling pathway in pulmonary fibrosis. IGF-1 is produced by alveolar macrophages (AMs) and fibroblasts. IGF-1 activates fibroblast via PI3K/Akt and MAPK signaling and leads to fibroblast-myofibroblast transition via PI3K/Akt, MAPK, EGR1/2, and FOS signaling. Transition of type II alveolar epithelial cells to fibroblast-like cells is mediated by IGF-1-induced ERK activation. IGF-1 mediates alternative activation of alveolar macrophages, generating type 2-activated macrophages. Except for fibroblasts and myofibroblasts, EMT-generated fibroblast-like cells and alternative type 2-activated macrophages are all capable of producing extracellular matrix (ECM) and profibrotic signals.

Despite these evidences, a recent study investigated the role of IGF-1R in experimental pulmonary fibrosis by exposing IGF-1R deficiency mice to bleomycin [146]. IGF-1R deficiency is associated with transcriptome changes, diminished Igf1 levels, and increased levels of Igfbp3, Igfbp5, Insr, and Foxo1 [146]. IGF-1R depletion improves mouse survival, reduces alveolar damage and HIF1A expression, protects against lung vascular fragility and permeability, and reduces lung inflammatory cell infiltration and bone marrow neutrophilopoiesis [146]. Cellular responses induced by IGF signaling pathway in pulmonary fibrosis are described in Figure 2.

## 5. IGF in Acute Lung Injury (ALI) and Acute Respiratory Distress Syndrome (ARDS)

ARDS, the most severe form of ALI, is one of the most fatal lung diseases [147]. IGF signaling was first identified as biomarkers for ALI and ARDS. In small case series, serum free IGF‐1 level is significantly elevated in the early ALI/ARDS group compared to that in healthy controls and fibroproliferative ARDS group. Free IGF‐1 levels in epithelial lining fluid, as well as lung IGF-1 mRNA expressions and IGF-1 immunoreactivity in lung biopsy specimens, are increased in fibroproliferative ARDS compared to those in healthy controls [148]. IGF-1 and IGF-1R immunoreactivity is enhanced in fibroproliferative ARDS and is seen in alveolar macrophages and epithelial cells lining the airways as well as in a variety of interstitial cells [148]. However, AMs collected from BALF of fibroproliferative ARDS are not elevated as compared with those of controls, which suggests lung cells other than AMs are the major source of IGF-1 expression in fibroproliferative ARDS [149].

The levels of IGF-1 and IGFBP-3 are elevated in at-risk patients and those with early ARDS, when epithelial damage and death occur, and decreased in late ARDS [150]. Antibody blockage of IGF-1R induces dose-dependent apoptosis of primary human lung fibroblasts but not primary lung epithelial cells or human macrophage cells [150]. ARDS is strongly associated with IGF-1 and IGFBP-3 levels in critically ill patients [151]. A rs2854746 polymorphism is significantly associated with plasma IGFBP-3 [151]. According to a large prospective case-control study, baseline plasma levels of IGF-1 and IGFBP-3 are significantly lower in ARDS cases than controls [152]. Among ARDS cases, IGF-1 and IGFBP-3 levels were significantly lower in nonsurvivors than survivors, and both are negatively associated with hazard of 60-day mortality in multivariate models [152]. Dynamic bioinformatic assay revealed that plasma IGF-1 level is higher in day 1, but lower in day 3 in severe pneumonia-associated ARDS than that in severe pneumonia [153]. Plasma level of IGF-1R is higher in days 1, 3, and 7 in severe pneumonia-associated ARDS than that in healthy controls [153]. Levels of IGFBP-4 and IGFBP-2 are also changed dynamically through the time course of ARDS, providing them the potential of being as a biomarker [153]. These inconsistent clinical data indicate the complexity of lung, muscle, and systemic inflammatory responses of ALI that may be time-dependent, organ-dependent, and individualized and may reflect the status of growth hormone resistance in ARDS [152–154]. In addition to extensive lung inflammation, aerosolized LPS in mice leads to increases in the numbers of airway epithelial cells and mucus cells and mucus production in a dose-dependent manner [155]. Hyperplastic epithelial cells are IGF-1-positive and expressing high levels of Bcl-2 and Muc5ac in this model [155]. IGF-1 knockdown suppresses Bcl-2 expression in human and murine airway epithelial cells and Muc5ac in primary murine airway epithelial cells [155]. Intranasally LPS challenge in mice induces IGF-1, Bcl-2, IL-1, and MUC5ac expression while it downregulates IGFBP-3 in laser-capture microdissected AECs by microarray analysis [156].

The production and absorption of alveolar edema in ALI and ARDS are mediated, respectively, by increased alveolar and vascular permeability and inactivation of epithelial sodium channel (ENaC), which are both regulated by IGF-1 signaling [157, 158]. Subcutaneous injection of recombinant human IGF-1 increases capillary permeability of the skin and the retina in healthy subjects [159]. IGF-1 improves the barrier properties of the aging microvascular endothelial cells* in vitro* and in ischemic mice brains [160]. IGF-1R knockout renders endothelial cells more prone to endothelial barrier dysfunction both* in vivo* and* in vitro*, possibly by disrupting the interaction between VE-protein tyrosine phosphatase and VE-cadherin [161]. IGF-1R signaling also activates vascular endothelial growth factor (VEGF) signaling via MAPK and has thus a protective effect in enhancing endothelial repair, regeneration, and vascularization [162, 163]. Intraocular, but not systemic expression of IGF-1 increases IGF-1R content and signaling and led to accumulation of VEGF and increased retinal vessel paracellular permeability [164].

LPS challenge in alveolar epithelial cells significantly downregulates *α*ENaC mRNA by reducing *α*ENaC promoter activity via the ERK1/2 and p38 MAPK pathways [165]. IGF-1 signaling regulates the activity of Nedd4, which is the key modulator of ubiquitinylation and subsequent degradation of ENaC [166]. This effect is mediated by phosphorylation of Akt1 and activation of Sgk-1 in primary rat fetal alveolar epithelial cells [167]. The ATII-protective effect of IGF-1 is also recorded, as intracheal injection of recombinant adenoviruses 5 of IGF-1 (Ad5-IGF-1) protects ATII cells and prevents mice from acute lung injury induced by perfluoroisobutylene inhalation [168]. Mesenchymal stem cell- (MSC-) derived conditioned medium ameliorates lipopolysaccharide- (LPS-) induced lung injury, by attenuating lung inflammation and promoting a wound healing/anti-inflammatory M2 macrophage phenotype, possibly via paracrine mechanisms involving IGF-1 [169]. These data in sum suggest a pathological and a potentially therapeutic role of IGF-1 in ALI and ARDS.

## 6. IGF in Asthma

### 6.1. Abnormal IGF-1 Signaling in Asthma

As has been reviewed, IGF-1 signaling pathway and IGFBP-3 are of therapeutic significance in asthma [170]. Although prenatal parental cigarette smoking may alter fetal growth and IGF-1 signaling [44–46], it is noteworthy that prepubertal allergic boys typically present with normal serum levels of IGF-1 and IGFBP-3 [171]. Thus data are insufficient for reaching the conclusions that congenital or prenatal IGF-1 signaling abnormality contributes to the development of asthma. IGF-1 mRNA level is significantly elevated in endobronchial biopsies from asthma patients and is correlated with subepithelial fibrosis, which is partly reversed by inhaled beclomethasone dipropionate [172]. Being as the basis for asthma treatment, steroids appear to inactivate IGF-1/IGF-1R signaling indirectly or directly by downregulating the expression of IGF-1 and IGF-1R both locally and systemically [173, 174]. Both dexamethasone and montelukast attenuate ovalbumin-induced asthmatic lung inflammation in guinea pigs, possibly via inhibition of IGF-1 and other proinflammatory signals [175]. Aerobic training, a recommended adjuvant treatment for asthmatic patients, reduces the expression of proinflammatory signals including IGF-1 and activation of peribronchial leukocytes, resulting in decreased airway inflammation and Th2 response in a mouse model of allergic asthma [176, 177]. In contrast, IGF-1 is among the proinflammatory mediators upregulated by creatine supplementation that exacerbates goblet cell proliferation and IL-5 and iNOS expression by epithelial cells in an asthma model [178, 179].

Genome-wide association study (GWAS) reveals that bronchodilator response in asthmatic children is associated with PAPPA2, which may cleave IGFBP-5 and regulate local IGF bioavailability [180, 181]. Microarray analysis reveals that IGF-1 mRNA is elevated in lungs from ovalbumin-induced asthmatic model, which could be reduced by knockout of retinoid-related orphan receptor alpha (ROR*α*) [182].

### 6.2. IGF-1 Signaling Activates Airway Smooth Muscle Cells in Asthma

IGF-1 stimulates mitogenesis of human airway smooth muscle (ASM) cells but not human fetal lung fibroblasts [183]. The production of granulocyte macrophage colony stimulating factor (GM-CSF), a recognized cytokine that maintains the inflammation and vascular leakage in asthma, is potentiated by IGF-1 in human ASM cells via p38 MAPK [184, 185]. ASM cells express IGF-1, IGF-2, IGF receptors, IGFBP-3, and IGFBP-2 [186]. IGFBP-2 that is secreted into the conditioned medium by ASM cells blocks the IGF-induced ASM proliferation [187]. IGF-1 is associated with enhanced proliferation and hyperplasia of ASM cells and induces Rho-kinase-dependent sustained contraction of human ASM cell rings [188]. The mitogenic effect of IGF-1 is enhanced by leukotriene D4 (LTD4) [189]. The synergetic effect of IGF-1 and LTD4 in ASM proliferation is mediated by MMP-1, which is one of the IGFBP proteases, thus enhancing IGF-1 activity [190]. Airway tissues from asthmatic patients show that the expression and proteolytic activity of MMP-1 are significantly enhanced in ASM cells and that IGFBP-2 and IGFBP-3 exist as cleaved forms in the airway tissues [189, 190].

TGF-*β*1-mediated stimulation of ASM cell proliferation is independent of IGF-1 signaling but is associated with significant increase in phosphorylated p38 MAPK, ERK1/2, and JNK, which is inhibited by selective inhibitors of p38 MAPK and MAP kinase kinase (MEK) but not by IGF-1 antibody [191]. Both IGFBP-3 and TGF-*β*1 have been separately shown to have cell-specific growth-inhibiting or growth-potentiating effects. Addition of IGFBP-3 or TGF-*β*1 stimulates ASM cell growth* in vitro*. However, the stimulatory effects of TGF-*β*1 on ASM cells seem to be mediated by IGFBP-3 in an IGF-1-independent manner. TGF-*β*1 upregulates IGFBP-3, but not IGFBP-2 or IGFBP-4, peptide expression in ASM cells. TGF-*β*1-stimulated ASM cell growth could be blocked by IGFBP-3 antisense oligomers as well as with an IGFBP-3 neutralizing antibody [192].

### 6.3. IGF-1 Signaling Activates Noncanonical Phagocytosis in Asthma

A recent study discloses novel roles of IGF-1/IGF-1R signaling in asthma [193]. The phagocytic engulfment capacity allows cells to uptake larger apoptotic cells and enhance internalization of smaller particles, which may influence inflammatory responses. Phagocytic activity of fibroblasts, airway epithelial cells, and endothelial cells are dampened* in vitro* by IGF-1 by phosphorylation of Akt and ERK and are further partly recovered by addition of IGF-1R inhibitors. Further* in vivo* experiments show that IGF-1 inhibits engulfment by airway epithelial cells but not AMs in mice, which may partly be attributed to relatively more prominent IGF-1R expression in airway epithelial cells than in AMs. In addition, IGF-1 production is inducible predominantly by AMs* in vivo* following intranasal administration of IL-4 or IL-13. Moreover, airway epithelial-specific depletion of Igf1r before, but not after, house dust mite exposure, renders mice more asthma-prone than control mice, as manifested by increased airway hyperresponsiveness, more extensive apoptosis and eosinophil, and CD4+ T cell infiltration in the lungs. These results reveal the role of IGF-1/IGF-1R in asthma by regulating phagocytosis and communication of AMs and airway epithelial cells.

In a more recent study, inflammatory responses in mice with nonorgan specific depletion of Igf1r are less prominent than their controls [194]. Igf1r-deficient mice exhibit no airway hyperresponsiveness, and a selective decrease in blood and BALF eosinophils, lung IL-13 levels, collagen, and smooth muscle, as well as a significant depletion of goblet cell metaplasia and mucus secretion markers after home dust mite exposure. The Igf1r-deficient mice displayed a distinctly thinner epithelial layer than control mice. There are little data to explain the contrary responses to allergen challenge in backgrounds of nonorgan specific- or alveolar epithelial cell-specific Igf1r depletion. However, sexual and systemic compensatory mechanisms may be involved, as in the latter study female mice are used and elevated Igf1 mRNA expression is documented.

### 6.4. IGF-1 Activates Immune Cells in Asthma

Additional data put forward the immunoregulatory role of IGF-1 in asthma, such as sensitizing immune cells including basophils, eosinophils, B lymphocytes, regulatory T cells, platelets, and stimulating the production of Th2 cytokines. IGF-1 promotes the expression of IL-13 in C5a-primed human basophils [195]. IGF-1 stimulates degranulation and histamine release of human basophils in response to IgE or Fc*ε*RI-mediated stimulation [196]. Recombinant IGF-1 stimulates chemokinesis and inhibits apoptosis selectively in basophils rather than in eosinophils, neutrophils, or monocytes [197]. IGF-1 induces IgE and IgG4 production by human B lymphocytes in an IL-4- and IL-13-dependent mechanism [198]. IGF-1 increases mRNA and protein expression of the IL-4-induced type II IgE receptor (Fc*ε*RII/CD23) in B cells, suggesting its immunomodulatory potential in asthma [199]. Studies revealed that T lymphocytes could be stimulated by Th2 cytokines such as IL-4, IL-9, IL-13, and IL-15, via IRS-1/IRS-2 signaling independent of IGF-1/IGF-1R [200–202]. Recombinant human IGF-1 directly stimulates proliferation of both human and mouse regulatory T cells (Treg)* in vitro*, which has been shown to regulate allergic responses in asthma [203]. Platelets play a significant role in asthma through miR-223-induced, advanced glycation end product-mediated vascular endothelial cell apoptosis via decreasing IGF-1R [204].

Positive feedback between IGF-1 and Th2 cytokines has been documented in asthma, as IGF-1 is inducible by repetitive intranasal challenge with IL-25 or IL-33 in murine asthma models [205, 206]. Another putative mediator of severe asthma, IL-17F, has been shown to induce IGF-1 expression in bronchial epithelial cells along or by costimulation with IL-4 and IL-13, suggesting an important relationship among IGF-1 signaling, Th2, and Th17 cells in asthma [207].

### 6.5. IGF-1 Signaling and Airway Remodeling

Airway inflammation and obstruction are induced by transition of airway epithelial cells and activation of airway fibroblasts, in which data are inconsistent regarding IGF-1. Bronchial wall remodeling is accompanied by increased number of myofibroblasts beneath the bronchial epithelial basement membrane. IGF-1 increases after epithelial damage, which may enhance myofibroblast proliferation in a paracrine manner, in the coculture system of primary human bronchial myofibroblasts and a bronchial epithelial cell line [208]. Previous studies also reveal IGF-1 as a chemoattractant for migration of bronchial epithelial cells* in vitro *[209]. However, there are also evidences showing that TGF-*β*1 induces EMT in bronchial epithelial cells via PI3K/Akt signaling independent of IGF-1 [210]. Migration of airway epithelial cells could be stimulated by IL-4, possibly via IRS-1/IRS-2 signaling independent of IGF-1/IGF-1R in an asthma model [211].

### 6.6. Targeting IGF-1 and IGFBP-3 in Animal Models of Asthma

Administration of an IGF-1 neutralizing antibody alleviates asthmatic outcomes including airway inflammation, subepithelial fibrosis, and elevated airway resistance in ovalbumin-challenged mice [193, 212, 213]. Expression of intercellular adhesion molecule-1 (ICAM-1) is also decreased in a dose-dependent manner without changing the levels of IL-4, IL-5, and IL-13 [213]. The expression of IGFBP-3 is high in asthmatic airway epithelial cells in situ and is increased in BALF of patients with asthma after allergen challenge. IGFBP-3 expression is upregulated by IGF-1 or TGF-*β*1 in primary airway epithelial cells [214]. Administration of IGFBP-3 or IGFBP-3 gene transfer attenuates ovalbumin inhalation-induced inflammatory cell recruitment, airway hyperresponsiveness, airway thickening, serum ovalbumin-specific IgE, vascular permeability, and Th2 cytokines (IL-4, IL-5, IL-13) production in mice lungs [213, 215]. IGFBP-3 also reduces IGF-1 expression, and blockade of IGF-1 with an neutralizing antibody is also shown to inhibit ovalbumin-induced VEGF expression, airway inflammation, and airway hyperresponsiveness [213].

**Figure 3:**
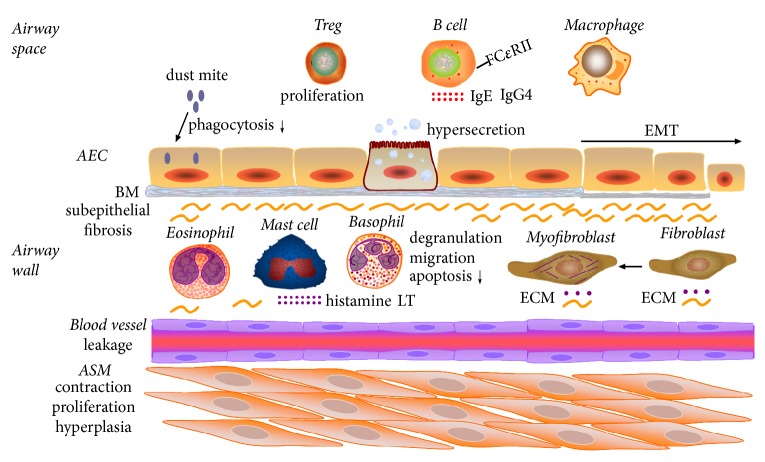
Cellular responses activated by IGF-1 in asthmatic airway inflammation. IGF-1 reduces AECs (airway epithelial cells) phagocytosis of dust mite and induces its hypersecretion, migration, and epithelial-mesenchymal transition (EMT). IGF-1 activates the proliferation of ASM (airway smooth muscle cell) and induces its contraction and hyperplasia. IGF-1 enhances blood vessel permeability. IGF-1 induces the degranulation of mast cell and basophil and release of histamine and leukotriene, activates the migration of basophils, and inhibits apoptosis. IGF-1 induces FC*ε*RII expression and production of IgE and IgG4 in B cells. IGF-1 activates the proliferation of Treg (regulatory T cells) and activates eosinophils and macrophages. IGF-1 activates fibroblasts and myofibroblasts which generates extracellular matrix (ECM). IGF-1 activates GM-CSF, VEGF, LTD4, IL-4, IL-5, IL-13, IL-25, IL-33, IL-17A, and vice versa. BM: basal membrane.

Whereas IGFBP-3 seems to regulate asthmatic inflammation through inhibition of IGF-1, a non-IGF-binding IGFBP-3 mutant shows similar results. These data highlight otherwise IGF-1-independent mechanisms of IGFBP-3, which may include inactivation of ovalbumin-activated VEGF and NF-*κ*B signaling [213, 215]. IGFBP-3 suppresses VEGF production by both IGF-1-dependent and hypoxia-inducible factor- (HIF-) dependent mechanisms, while VEGF has been shown to be associated with subepithelial fibrosis by regulation of TGF-*β*1 expression through the PI3K/AKT signaling pathway in asthma [213, 216]. IGFBP-3 inactivates NF-*κ*B signaling pathway in asthma in that it degrades I*κ*Ba and p65-NF-*κ*B through IGFBP-3 receptor-mediated, IGF-1-independent activation of caspases, which further blocks TNF-*α*-induced inflammation and eosinophil migration [215]. The cellular responses modified by IGF-1 signaling in asthma are depicted in Figure 3. 

## 7. IGF-1 Signaling in COPD

### 7.1. IGF-1 Signaling in Inflammations of COPD

COPD has been redefined as a disease with both lung inflammation and systemic inflammatory responses [217]. Abnormal IGF-1 signaling is present in most studies except one, which shows that circulating levels of IGF-1 are similar in patients with stable COPD as compared to control levels [218]. Other studies show that the serum levels of IGF-1 are significantly lower in COPD patients than in healthy controls, which are associated with the disease severity but not with peripheral neuropathy [219–222]. The serum levels of IGF-1 are significantly decreased in patients admitted for acute exacerbations of COPD (AECOPD) compared with those in stable stage COPD patients [219, 223, 224] and then increase partly at the time of patients' discharge, but the serum levels of IGF-1 both on admission and on discharge of AECOPD patients are lower than those of healthy controls [223, 224]. Emphysematous patients seem to have significantly lower IGF-1 levels compared to those with chronic bronchitis both on admission and at discharge [223].

A reasonable explanation may be that cytokines produced excessively in AECOPD disturb IGF-1 production, as 24 weeks of infliximab (a TNF-*α* antibody) treatment reduces serum levels of IGF-1 in COPD patients and TNF-*α* or IL-1 suppresses IGF-1 bioactivity via induction of IGFBP-1 synthesis both* in vitro* and* in vivo *[225, 226]. In the study by Kythreotis et al. however, serum levels of IGF-1 are not related to levels of TNF-*α*, IL-1, IL-6, and IL-8 neither on admission nor at discharge [223]. Involvement of alternative mechanisms is thus implicated. IGF-1 signaling pathway may control the growth and fate of several lung cells in emphysema. IGF-1/IGF-1R signaling increases Bcl-2 mRNA expression and stability in airway epithelial cells* in vitro* via convergence of IGF-1R and EGFR signaling. Colocalization of IGF-1 and Bcl-2 is induced in airway epithelial cells by cigarette smoking in mice or is observed in lung tissues from patients with chronic bronchitis [156]. The protein and mRNA expression of IGFBP-3 are inducible in alveolar epithelial cells exposed to cigarette smoke, which could be attenuated by pretreatment of sulforaphane, an antioxidant agent. Sulforaphane decreases intracellular ROS, restores the viability of A549 cells, and attenuates G1 block of the cell cycle in cigarette smoke-exposed A549 cells [227]. Primary lung fibroblasts harvested from patients with emphysema are senescent, which express higher levels of IGFBP-3 mRNA and protein than those from controls [228, 229]. The possible prosenescence effect of IGFBP-3 in fibroblasts is supposed to be mediated by IGFBP-3/IGF-2 interactions, not by IGF-1 [230].

### 7.2. IGF-1 Signaling in Muscle Dysfunction and Metabolism of COPD

Along with overall impaired muscle strength and endurance, skeletal muscles are characterized by fibrosis and thinner muscle fibers, while necrosis, mitochondrial abnormalities, and inflammatory cell infiltration are the same in COPD patients compared to controls [231]. In some COPD cases respiratory muscles are also impacted and even cachexia develops, in which IGF-1 signaling plays an important role [232]. However, the expression trends of muscle IGF-1 mRNA or protein are inconsistent between studies. Quadriceps of IGF-1 mRNA are increased in COPD patients compared with controls [233]. IGF-1 is positively correlated with indicators of malnutrition and muscle wasting, including body weight, body mass index, thigh circumference, and albumin level [220]. The strength of quadriceps decreased in AECOPD is positively correlated with serum levels of IGF-1 [234]. Igf1 knockout reduces the thickness and the number of fibers of mice diaphragm [235]. IGF-1 mRNA levels are decreased in biopsies of vastus lateralis from hospitalized AECOPD patients [236]. Circulating IGF-1 levels are similar in cachectic versus noncachectic COPD patients [237]. However, muscle IGF-1 protein is decreased more in cachectic COPD patients compared with noncachectic patients [238]. Testosterone and/or resistance training strengthens quadriceps muscle synthesis in COPD patients by increasing local IGF-1 expression [239].

Pulmonary rehabilitation induces mRNA and protein expressions of IGF-1 in vastus lateralis, which are associated with improvement of muscle quality and strength in COPD patients [240]. IGF-1 protein levels in quadriceps increase more in noncachectic patients than in cachectic COPD patients after pulmonary rehabilitation training [238]. Improved six-minute walk test performance is associated with increased levels of serum IGF-1 and IGFBP-3 following pulmonary rehabilitation in COPD patients [241]. The roles of IGF-1 in muscle metabolism are further proved in animal models of chronic cigarette smoke exposure. Compared to controls, rodents chronically exposed to cigarette smoke are presented with significantly lower plasma IGF-1 levels and decreased IGF-1 mRNA and protein expression in the gastrocnemius, which may be associated with reduced body weight and food intake, systemic inflammation, skeletal muscle wasting, and reduced skeletal muscle strength [242, 243]. Exogenous rhIGF-I attenuates diaphragm fiber atrophy induced by triamcinolone administration in a hamster model of pancreatic elastase-induced emphysema and prevents them from significant weight loss without interfering blood glucose levels [244]. Administration of rhIGF-1 completely prevented atrophy and growth arrest of all diaphragm fibers in rats with moderate caloric deficit [245]. In animal studies of chronic hypoxia exposure, recombinant human IGF-1 significantly ameliorates protein catabolism and promotes anabolism, increases serum total protein and albumin, and earns greater weight gain than vehicle [246, 247]. Notably, hypoxia and caloric deficit are common in COPD patients which may exacerbate muscle dysfunction [248].

### 7.3. IGF-1 Signaling and Aging in COPD

IGF-1 signaling pathway has been proposed as a target for aging-related diseases [249, 250]. As accelerated lung aging features COPD, conquering aging becomes a promising therapeutic route for COPD [251]. Direct evidences for IGF-1 signaling in aging are also shown. IGF-1 knockdown protects mice from paraquat-induced oxidative stress and death and prolongs mice lifespan by inhibiting MAPK/ERK1/2 and Akt signaling [252]. IGF-1 protects from DNA injury and facilitates repair [253]. Excessive IGF-1 inhibits autophagy via Akt/mTOR signaling in cigarette smoke extract-treated human bronchial epithelial cells, which further modulates cell senescence and contributes to the pathogenesis of COPD [254]. IGFBP-3 may also regulate senescence of alveolar epithelial cells and lung fibroblasts in emphysema [223–225]. These data offer IGF-1 signaling pathway as a potential target for aging in COPD [255, 256].

## 8. IGF-1 Signaling in Cystic Fibrosis (CF)

CF is caused by mutations in the gene that encodes cystic fibrosis transmembrane conductance regulator (CFTR) protein [270]. CFTR protein is an epithelial chloride channel that regulates transepithelial transport of sodium and bicarbonate. This leads to a collection of systemic manifestations, which involves the pulmonary, digestive, reproductive, and endocrine system [270–272]. Ninety percent of CF patients die for progressive lung disease, and airway inflammation is an important feature for CF [270, 273]. Both lung inflammation and systemic responses in CF patients are associated with inhibited IGF-1 signaling. CF patients have significantly lower serum and BALF levels of IGF-1 compared with health controls [257–261] and higher serum levels of IGFBP-2 [260]. Low serum levels of IGF-1 and high levels of serum IGFBP-2 are associated with increased IL-1*β*, IL-6, and TNF-*α* levels [260]. The number of IGF-1 and Bcl-2 double-positive AECs is elevated in lung specimens from CF patients, which suggests an antiapoptotic effect of IGF-1, possibly mediated by PI3K/Akt signaling [156]. IGF-1 expression is accompanied by increased numbers of myofibroblasts, collagen I deposition, alveolar inflammation, and elastin degradation in CF lung tissues [258]. Serum IGF-1 levels fall significantly in pulmonary exacerbation of CF from baseline, which might be improved further by intravenous antibiotic treatment [257]. IGF-1-positive staining in AECs and macrophages are distributed in lung tissue specimens from CF patients with* Pseudomonas aeruginosa *colonization prior to lung transplantation [258]. AMs isolated from BALF of CF patients exhibit impaired phagocytosis activity in culture with* Pseudomonas aeruginosa*, which could be dose-dependently improved by exogenous IGF-1 [259].

Notably, pigs, mice, or humans with CF are born with reduced serum insulin-like growth factor 1 (IGF-1) levels [262, 263]. There are several potential mechanisms underlying downregulation of IGF-1 signaling in CF. CFTR deficiency activates sulfotransferase 1E1 (SULT1E1), which inhibits IGF-1 synthesis by inactivation of beta-estradiol at physiological concentrations via conjugation with sulfonate [274, 275]. On the other hand, IGF-1 enhances stable CFTR expression and CFTR-mediated chloride transport in CF cell lines, which depends on activation of TC10 and inactivation of CFTR-associated ligand (CAL) [276]. IGF-1-activated TC10 changes the interaction of CFTR and CAL in the Golgi, allowing CFTR to progress to the plasma membrane instead of trafficking to the cell surface for degradation [276, 277]. Taking these data together, CFTR deficiency and decreased IGF-1 signaling constitute the positive feedback that exacerbate each other. Furthermore, IGF-1 may be regulated by IGFBPs, GH, and insulin metabolism. Serum IGFBP-3 levels might be decreased [278], while IGFBP-2 levels may increase in CF patients [259]. Normal spontaneous and stimulated GH levels but low levels of IGF-1 and IGFBP-3 suggest a state of relative GH insensitivity in children with CF [279, 280]. There is also a significant association between plasma insulin area under curve and serum IGFBP-3, and patients whose insulin secretion is most impaired had lower serum IGFBP-3 levels but higher IGFBP-1 levels than normal controls [276]. Thus insulin deficiency may disturb the GH/IGF-1 axis as a result of reduced levels of IGFBP-3 and increased levels of inhibitory IGFBP-1 [281, 282].

Reduced IGF-1 signaling has endocrine and metabolic implications in CF. Progressive insulin deficiency may reduce IGF-1 levels and IGF-1 bioactivity and disturb weight gain and statural growth in CF [281]. Serum IGF-1 levels are directly correlated with FEV1%, serum iron, hemoglobin concentration, and transferrin saturation [257–259, 264]. Both IGF-1 and IGFBP-3 levels are correlated with height, weight, BMI, and protein catabolism, which reflect growth retardation in prepubertal CF patients [265, 266, 278–280]. In an animal model of CF, serum Igfbp-3 levels are increased, while Igf-1, albumin, and triglycerides measures are decreased compared with wild-type mice [280]. Recombinant human IGFBP-3 treatment significantly increased serum albumin and triglycerides but did not affect weight gain in CF mice [283]. Except for intertwining with GH and insulin, IGF-1 may also regulate metabolism by upregulating the depressed expression of FOXO1 in CF [284].

Since IGF-1 and GH are downregulated and associated with inflammation, metabolism, and development in CF patients, supplement of exogenous IGF-1 or GH seems to be a rational therapy. Administration of recombinant growth hormone significantly increases height, growth velocity, and serum levels of IGF-1 and IGFBP-3, but not lung functions [285]. Meta-analyses show that growth hormone therapy improves the intermediate outcomes in height, weight, and lean tissue mass, but pulmonary function improvement is inconsistent [286]. No significant changes in quality of life, clinical status, or side-effects are observed [287]. Both commercialized rhIGF-1 (mecasermin) and recombinant human IGFBP-3 have been available and approved by the U.S. Food and Drug Administration (FDA) for over 10 years [288, 289]. In a clinical trial, treatment with IGF-1 significantly increases serum IGF-1 levels in prepubertal children with CF but does not result in significant difference in linear growth rate, weight gain, rate of accretion of lean body mass, or mean FEV1 as compared with placebo [267]. It is argued that higher dose of IGF-1 treatment may bring about higher efficacy in CF, but insulin sensitivity might be impaired as well [288]. In a case report, prolonged use of rhIGF-1 significantly improves body growth, stabilizes lung function, and reduces the need of insulin for glycemic control in a child with CF [267]. This indicates the long-term safety and efficacy of rhIGF-1 for patients with CF-associated growth failure. However, data are insufficient with regard to lung and systemic inflammations before and after long-term rhIGF-1 administration. A prospective study was terminated for insufficient recruitment, which aims to investigate the efficacy of rhIGF-1 on body weight and composition within 28 weeks in CF adults [268]. rhIGF-1 treatment is thus promising for restoring GH/IGF-1 axis, metabolism, and growth in CF patients, but its efficacy in lung inflammation and immunology awaits further investigations.

There are concerns that IGF-1 might have roles in non-CF bronchiectasis like in CF. However, there are currently no direct evidences for or against this. According to Jian et al., 46% RA-ILD patients have bronchiectasis on HRCT scan, and serum levels of IGF-1 are increased in RA-ILD compared to those in RA and control subjects [89]. The prevalence of mild cylindrical bronchiectasis is 22.2% in 36 patients with acromegaly but is not statistically different from 24 control subjects (16.7%) and is not associated with serum levels of IGF-1 (*P* = 0.74) [41]. Another study by Camilo et al. shows 35% of 20 patients with acromegaly have bronchiectasis, but serum IGF-1 levels are not statistically associated with lung function changes [290]. Taken these data together, rheumatoid arthritis- or acromegaly-assicated bronchiectasis is not associated with changes of serum IGF-1 levels. Despite the similarities of CF and non-CF bronchiectasis, there are currently no evidences supporting the supplemental administration of rhIGF-1 in stable bronchiectasis or acute exacerbations of bronchiectasis [291].

## 9. Conclusions and Future Directions

In this article, we reviewed the up-to-date roles and possible mechanisms of IGF-1 signaling pathway in lung development and inflammatory diseases including BPD, pulmonary fibrosis, ALI and ARDS, asthma, COPD, and CF. As abnormal IGF-1 signaling disrupts alveolar development, it is even more important to know how IGF-1 signaling manipulates different cells in the precise time and space sequence and its crosstalk with other signaling pathways, both in normal and in abnormal circumstances. Whereas components of IGF-1 signaling pathways are potentiated as biomarkers as they are dysregulated locally or systemically in inflammatory lung diseases, data may be inconsistent or even be paradoxical among different studies (summarized in Table 1). This could be partly attributed to the complexity of IGF-1, which serves both as an important part of the growth hormone/IGF-1 axis and also as a regulator of systemic metabolism. And the molecular-, time-, and organ-dependent and individualized IGF-1 signaling further obscures the potentials of its components for being candidate biomarkers for lung diseases [154]. Moreover, IGF-1/IGF-1R antibodies are well tolerated in clinical trials but fail to generate overall therapeutic advantages in patients with small cell lung cancer or in patients with non-small cell lung cancer by combination with chemotherapy or tyrosine kinase inhibitors (TKIs) [12–16, 269]. These unfavorable outcomes include retraction of earlier publications of a phase II trial data, following a thorough investigation of the previously reported survival results [292]. The usage of IGF-1-based agents urges for more researches in developmental disorders and inflammatory lung diseases, as current data are generally collected from limited number of animal experiments and are less exuberant than those in lung cancer. An exception is the successful usage of rhIGF-1 in a few CF cases, although rhIGF-1 seems to improve metabolism and development most. IGF-1 antibody, rhIGF-1, and rhIGFBP-3 are commercially available and under investigations for clinical efficacy in patients with lung diseases, as described in Table 2. Much is still awaiting elucidations regarding this issue that direct us towards further research directions. By integrating bench-bed research methods, rational clinical diagnostic and therapeutic approaches concerning IGF-1 signaling might become applicable in lung disorders in the next few years.

## Figures and Tables

**Table 1:** Changes and clinical relevance of serum IGF-1 levels in lung diseases.

	Changes	Clinical relevance	Ref.
BPD	Decreased	Low IGF-1 levels associate with the risk of developing BPD. A clinical trial (NCT01096784) showed the potential of supplement rhIGF-I/rhIGFBP-3 in reducing the prevalence of BPD in premature infants.	[69, 70]
IPF	Increased	Preclinical studies have shown that inhibition of IGF-1 signaling attenuates experimental pulmonary fibrosis.	[81–92, 146]
ALI/ARDS	Early stage: increased Late (fibroproliferative) stage: decreased	Low IGF-1 levels associate with the risk of developing ARDS and the mortality of ARDS patients. Preclinical studies have shown that IGF-1 attenuates experimental lung injury.	[150–154, 168]
Asthma	Increased	Preclinical studies have shown that lung-specific IGF-1R depletion renders mice more asthma-prone, while nonorgan specific IGF-1R protects mice from airway hyperresponsiveness and asthmatic airway inflammation.	[193, 194]
COPD	Decreased in stable stage and decreased more sharply in acute exacerbation	Associated with inflammation, skeletal muscle dysfunction, abnormal metabolism, and weight loss. Preclinical studies have shown that IGF-1 improves skeletal muscle synthesis and function.	[218–226, 231–245]
Cystic fibrosis	Decreased in stable stage and decreased more sharply in acute exacerbation	Associated with systemic/alveolar inflammation, airway infection, and relative growth hormone insufficiency; associated with growth retardation and metabolic abnormalities. Prolonged use of IGF-1 has shown efficacy in improving growth and metabolism in a CF child. A clinical trial of IGF-1 in CF patients was terminated for insufficient enrollment.	[257–268]
Lung cancer	Inconsistent data (increased or decreased)	Serum IGF-1 level is associated with the stage of lung cancer; predictive of resistance to EGFR-TKI therapy. Clinical trials of IGF-1/IGF-1R mAb/inhibitor have generated results; a few of the results are positive.	[10, 12–16, 269]
ARMS	Insufficient data	IGF-1/IGF-1R-based therapies are to be investigated in clinical trials; presumably drug resistance develops soon with IGF-1R mAb.	[17]

BPD: bronchopulmonary dysplasia. IPF: idiopathic pulmonary fibrosis. ALI/ARDS: acute lung injury/acute respiratory distress syndrome. COPD: chronic obstructive pulmonary disease. EGFR-TKI: epidermal growth factor receptor-tyrosine kinase inhibitor. ARMS: alveolar rhabdomyosarcoma.

**Table 2:** IGF-1-targeted agents and their trials in lung diseases.

Category	Agents	Lung diseases in clinical trials	Major results or study design of representative trials
rhIGF-1	Mecasermin (Increlex), Myotrophin, CEP-151	Cystic fibrosis	NCT00566241: rhIGF-1 versus placebo on body weight and body composition in CF patients [terminated for insufficient recruitment of participants].

rhIGF-1/rhIGFBP-3	Mecasermin rinfabate (Iplex)	BPD	NCT01096784: rhIGF-I/rhIGFBP-3 versus standard care in the occurrence of BPD in premature infants: 21.3% (10/47) versus 44.9% (22/49) (prevention of BPD is one of the secondary outcomes in this study).

IGF-1R TKI	Linsitinib (OSI-906, ASP7487)	Lung cancer	NCT01533181: linsitinib versus topotecan in relapsed or recurrent SCLC; PFS^*∗*^: 1.2 versus 3.0 m; OS^#^: 3.4 versus 5.3 m. *Leighl et al. [15]*: linsitinib + erlotinib versus erlotinib in chemotherapy-naive patients with EGFR-mutation positive advanced NSCLC; PFS^#^: 8.4 versus 12.4 m; ORR^*∗*^: 47.7% versus 75.0%; disease control rate^*∗*^: 77.3% versus 95.5%.
	AXL1717	Lung cancer	NCT01561456: AXL1717 versus docetaxel in SCC or lung adenocarcinoma. NCT01466647: noncontrolled trial of AXL1717 + gemcitabine/carboplatin in SCC. *Bergqvist et al. [14]*: AXL1717 versus docetaxel in patients with previously treated, locally advanced, or metastatic NSCLC; PFS^#^: 38.7 versus 37.4 weeks; rate of progression-free after 12 weeks: 25.9% versus 39.0%; ORR after 12 weeks^*∗*^: 0 versus 12.2%; disease control rate^#^: 24.1% versus 36.6%.
	XL228 [against IGF-1R, Src, FGFR, and BCR-Abl]	Lung cancer	NCT00526838: noncontrolled phase 1 trial of intravenous XL228 in subjects with advanced malignancies [terminated by the sponsor].

IGF-1R mAb	Figitumumab (CP-751, 871)	Lung cancer	NCT00596380: figitumumab + paclitaxel/carboplatin versus paclitaxel/carboplatin as first-line treatment for advanced nonadenocarcinoma NSCLC; PFS^#^: 4.7 versus 4.6 m; OS^#^: 8.6 versus 9.8 m; ORR^#^: 33% versus 35%. NCT00147537: figitumumab + paclitaxel/carboplatin versus paclitaxel/carboplatin as first-line treatment for advanced NSCLC; ORR^*∗*^: 37.4% versus 27.5%; PFS in groups of figitumumab 20 mg/kg, 10 mg/kg, or 0: 4.5 versus 4.4 versus 4.3 m. NCT00560573: figitumumab + gemcitabine/cisplatin versus figitumumab + pemetrexed/cisplatin as first-line treatment for advanced NSCLC; PFS: 6.5 versus 5.4 m; ORR: 56.5% versus 46.2%. NCT00673049: figitumumab + erlotinib versus erlotinib in advanced nonadenocarcinoma NSCLC; PFS^#^: 2.1 versus 2.6 m; OS^#^: 5.7 versus 6.2 m; ORR^#^: 5.5% versus 3.8% [terminated because combination therapy seemed to be unlikely to improve OS].
	Cixutumumab (IMC-A12)	Lung cancer	NCT00887159: cixutumumab + etoposide/cisplatin versus etoposide/cisplatin versus vismodegib (a Hedgehog pathway inhibitor) + etoposide/cisplatin in extensive stage SCLC; PFS: 4.6 versus 4.4 versus 4.4 m; OS: 10.1 versus 8.8 versus 9.8 m; ORR: 50% versus 48% versus 56%. NCT00986674: cetuximab + paclitaxel/carboplatin versus cixutumumab + paclitaxel/carboplatin versus cetuximab + cixutumumab + paclitaxel/carboplatin in patients with advanced NSCLC who will not receive bevacizumab-based therapy; PFS: 3.4 versus 4.2 versus 4.0 m; OS: 9.8 versus 7.7 versus 8.8 m; ORR: 11.1% versus 22.0% versus 21.7%; deaths within 1 month: 6 versus 2 versus 5 [terminated because of excessive grade 5 adverse events in patients receiving cetuximab therapy]. NCT00955305: paclitaxel/carboplatin + bevacizumab versus paclitaxel/carboplatin + bevacizumab + cixutumumab in advanced nonsquamous NSCLC; PFS^#^: 5.8 versus 7.0 m; OS^#^: 16.2 versus 16.1 m; ORR^#^: 46.2% versus 58.7% [terminated for accrual with cixutumumab]. *Novello et al. [269]*: pemetrexed/cisplatin + cixutumumab versus pemetrexed/cisplatin as first-line therapy in advanced nonsquamous NSCLC; PFS^#^: 5.45 versus 5.22 m; OS^#^: 11.33 versus 10.38 m; ORR^#^: 37.9% versus 30.6%.
	Dalotuzumab (MK-0646, h7C10)	Lung cancer	NCT00654420: erlotinib versus erlotinib + dalotuzumab in recurrent NSCLC; PFS^#^: 1.6 versus 2.5 m; OS^#^: 14.5 versus 6.9 m; ORR^#^: 5.6% versus 2.8%. NCT00799240: pemetrexed/cisplatin versus pemetrexed/cisplatin + dalotuzumab in stage IV metastatic nonsquamous NSCLC; 14 versus 12 participants; partial response: 2 versus 3; stable disease: 7 versus 4; progressive disease: 3 versus 4; not evaluable: 2 versus 1; median time to progression: 173 versus 199 d; median survival: 262 versus 276.5 d [terminated by the sponsors].
	Teprotumumab (R1507, RV-001)	Lung cancer	NCT00760929: teprotumumab (weekly) + erlotinib versus teprotumumab (per 3 week) + erlotinib versus placebo + erlotinib in advanced NSCLC with progression following one or two prior regimes; PFS^#^: 1.87 versus 2.7 versus 1.5 m; OS^*∗*^: 8.1 versus 12.1 versus 8.1 m; ORR: 8.8% versus 7% versus 8.8%. NCT00773383: teprotumumab + erlotinib in stage IIIB/IV NSCLC with progressive disease after clinical benefit to second- or third-line erlotinib monotherapy; rate of progression-free and alive in 3 weeks: 32.4%.
	Robatumumab (SCH 717454, MK-7454)	Solid tumors (including lung cancer)	NCT00954512: noncontrolled trial of robatumumab + paclitaxel/carboplatin in advanced NSCLC; 3 participants: 2 with stable disease and 1 with progressive disease; terminated for business reasons.
	Ganitumab (AMG-479)	Lung cancer, ARMS	NCT00819169: noncontrolled trial of ganitumab + conatumumab (a death receptor 5 agonist) in advanced solid tumors: nonsquamous NSCLC: stable disease 40%, progressive disease 60%, PFS 1.6 m; SCC: stable disease 71%, progressive disease 29%, PFS 3.3 m. NCT00791154: etoposide + platinum (carboplatin or cisplatin) ± ganitumab for extensive stage SCLC. NCT03041701: ganitumab + dasatinib (a Src family kinase inhibitor) in embryonal and alveolar rhabdomyosarcoma.
	BIIB022	Lung cancer	NCT00970580: BIIB022 + paclitaxel/carboplatin in treatment-naive advanced NSCLC.

IGF-1/IGF-2 mAb	BI 836845	Lung cancer	NCT02191891: BI 836845 + afatinib in patients with EGFR mutant NSCLC with progression following prior EGFR-TKI treatment; recruiting participants.
	MEDI-573	Solid tumors	No results reported.

IGF-1 inhibitor	Pasireotide (SOM230) [against somatostatin, IGF-1, GH, ACTH)	Lung cancer	NCT01563354: pasireotide versus everolimus versus pasireotide + everolimus in patients with neuroendocrine carcinoma of the lung and thymus. NCT01417806: pasireotide + topotecan in relapsed or refractory SCLC.

Other agents to be tested in lung disease trials	PL225B, AG-1024, AEW541, BVP51004 (kinase inhibitors of IGF-1R); BMS-754807, A-928605 (kinase inhibitors of IGF-1R and IR); KW-2450 (a pan-kinase inhibitor of IGF-1R, IR, Aurora A/B kinases); INSM-18 (a kinase inhibitor of IGF-1R and ErbB2); AVE1642 (an IGF-1R mAb); MM-141 (a mAb against IGF-1R and ErbB3); IGF-1 antisense oligodeoxynucleotide; IGF-methotrexate conjugate (765IGF-MTX); fusion protein of IGF-1 and vitronectin (VF001-DP); pUMVC3-IGFBP2-HER2-IGF1R plasmid DNA vaccine.		

BPD: bronchopulmonary dysplasia. SCLC: small cell lung cancer. NSCLC: non-small cell lung cancer. SCC: squamous cell carcinoma. EGFR-TKI: epidermal growth factor receptor-tyrosine kinase inhibitor. PFS: progression-free survival. OS: overall survival. ORR: objective response rate. ARMS: alveolar rhabdomyosarcoma. GH: growth hormone. ACTH: adrenocorticotropic hormone. ^*∗*^*P* < 0.05. ^#^*P* > 0.05. All the clinical trial data were accessed from PubMed or from the website ClinicalTrials.gov, which are services held by the U.S. National Institutes of Health. The authors declare no conflict of interests.

## Abbreviations

*α*-SMA: Alpha-smooth muscle actin
rhIGF-1: Recombinant human insulin-like growth factor-1
AEC: Alveolar epithelial cell
AECOPD: Acute exacerbation of chronic obstructive pulmonary disease
ALI: Acute lung injury
AM: Alveolar macrophage
ARDS: Acute respiratory distress syndrome
ASM: Airway smooth muscle
ATI: Type I alveolar epithelial cell
ATII: Type II alveolar epithelial cell
BALF: Bronchoalveolar lavage fluid
BPD: Bronchopulmonary dysplasia
CCSP: Clara cell secretory protein
CF: Cystic fibrosis
CFTR: Cystic fibrosis transmembrane conductance regulator
COPD: Chronic obstructive pulmonary disease
CTGF: Connective tissue growth factor
ECM: Extracellular matrix
EGFR: Epidermal growth factor receptor
EMT: Epithelial-mesenchymal transition
ENaC: Epithelial sodium channel
ERK: Extracellular signal-regulated kinase
FGF: Fibroblast growth factor
GH: Growth hormone
GM-CSF: Granulocyte macrophage colony stimulating factor
IFN-*γ*: Interferon-*γ*
IGF-1: Insulin-like growth factor-1
IGF-1R: IGF-1 receptor
IGF-2: Insulin-like growth factor-2
IGF-2R: IGF-2 receptor
IGFBP: IGF binding protein
IGFBP-rP: IGF binding protein-related protein
IPF: Idiopathic pulmonary fibrosis
IR: Insulin receptor
IRS: Insulin receptor substrates
JNK: C-Jun N-terminal kinase
LPS: Lipopolysaccharide
LTB4: Leukotriene B4
MAPK: Mitogen-activated protein kinase
MTOR: Mammalian target of rapamycin
NEDD4: Neural precursor cell-expressed developmentally downregulated protein 4
NF-*κ*B: Nuclear factor-*κ*B
PDGF: Platelet-derived growth factor
PI3K: Phosphoinositide-3-kinase
RTK: Receptor tyrosine kinase
SGK-1: Serum- and glucocorticoid-dependent kinase
SPC: Surfactant protein C
TGF-*β*: Transforming growth factor-*β*
TNF-*α*: Tumor necrosis factor-*α*
VEGF: Vascular endothelial growth factor
Wnt: Wingless/int1.
